# Analysis of MreB interactors in *Chlamydia* reveals a RodZ homolog but fails to detect an interaction with MraY

**DOI:** 10.3389/fmicb.2014.00279

**Published:** 2014-06-06

**Authors:** Scot P. Ouellette, Kelsey J. Rueden, Emilie Gauliard, Logan Persons, Piet A. de Boer, Daniel Ladant

**Affiliations:** ^1^Unité de Biochimie des Interactions Macromoléculaires, Département de Biologie Structurale et Chimie, Institut Pasteur, CNRS UMR 3528Paris, France; ^2^Division of Basic Biomedical Sciences, Sanford School of Medicine, University of South DakotaVermillion, SD, USA; ^3^Université Paris Diderot, Sorbonne Paris Cité, Cellule PasteurParis, France; ^4^Department of Molecular Biology and Microbiology, School of Medicine, Case Western Reserve UniversityCleveland, OH, USA

**Keywords:** *Chlamydia*, bacterial two-hybrid system, protein–protein interactions, cell division, RodZ, MreB

## Abstract

*Chlamydia* is an obligate intracellular bacterial pathogen that has significantly reduced its genome in adapting to the intracellular environment. One class of genes for which the bacterium has few annotated examples is cell division, and *Chlamydia* lacks FtsZ, a central coordinator of the division apparatus. We have previously implicated MreB as a potential substitute for FtsZ in *Chlamydia* (Ouellette et al., 2012). Thus, to identify new chlamydial cell division components, we searched for proteins that interacted with MreB. We performed a small-scale screen using a Gateway® compatible version of the Bacterial Adenylate Cyclase Two Hybrid (BACTH) system, BACTH_GW_, to detect proteins interacting with chlamydial MreB and identified a RodZ (YfgA) homolog. The chlamydial RodZ aligns well with the cytoplasmic domain of *E. coli* RodZ but lacks the periplasmic domain that is dispensable for rod cell shape maintenance in *E. coli*. The expression pattern of *yfgA/rodZ* was similar to that of *mreB* and *ftsI*, suggesting that these genes may operate in a common functional pathway. The chlamydial RodZ correctly localized to the membrane of *E. coli* but was unable to complement an *E. coli rodZ* mutant strain, likely because of the inability of chlamydial RodZ to interact with the native *E. coli* MreB. Finally, we also tested whether chlamydial MreB could interact with MraY, as suggested by Gaballah et al. (2011). However, we did not detect an interaction between these proteins even when using an implementation of the BACTH system to allow native orientation of the N- and C-termini of MraY in the periplasm. Thus, further work will be needed to establish this proposed interaction. In sum, we have added to the repertoire of potential cell division proteins of *Chlamydia*.

## Introduction

*Chlamydia trachomatis* is an obligate intracellular bacterial pathogen that causes blinding trachoma and sexually transmitted diseases (Schachter et al., 1973; Mabey et al., 2003). *Chlamydia* has a unique developmental cycle that alternates between an extracellular, infectious form (the elementary body or EB that mediates attachment to and uptake by susceptible host cells) and an intracellular, non-infectious form (the reticulate body or RB that grows and divides by binary fission) (see AbdelRahman and Belland, 2005 for review). A feature of *Chlamydia* intracellular growth is that it remains within a pathogen-specified vacuole, called an inclusion, for the duration of its developmental cycle. The inclusion remains non-fusogenic with the endolysosomal system but is highly interactive with host cell trafficking pathways.

One unusual aspect of chlamydial microbiology is the lack of a homolog for the essential cell division protein, FtsZ, a tubulin-like homolog (Mukherjee et al., 1993). However, chlamydiae do encode homologs for rod-shape determining proteins in spite of their coccoid shape (Ouellette et al., 2012). We recently hypothesized that MreB, an actin-like homolog critical in the establishment and maintenance of the rod shape of bacilli (Doi et al., 1988; Bork et al., 1992), may substitute for FtsZ in *Chlamydia* and presented evidence that chlamydial MreB is necessary for division of this bacterium (Ouellette et al., 2012).

MreB interacts with a number of proteins, one of which is YfgA. YfgA has been characterized as a rod-shape determining protein called RodZ (Shiomi et al., 2008; Alyahya et al., 2009; Bendezú et al., 2009). *E. coli* YfgA/RodZ contains a cytoplasmic region, which encodes a helix-turn-helix (HTH) domain that is important for interactions with MreB, a transmembrane domain, and a periplasmic domain (Bendezú et al., 2009; van den Ent et al., 2010). Very recently, a RodZ homolog in *Waddlia* (an organism related to the Chlamydiales), was implicated in division of that organism by recruiting MreB to the division plane (Jacquier et al., 2014). The authors suggested that peptidoglycan precursors are recruited to the division plane prior to MreB in part because MreB is purported to interact with components of the peptidoglycan enzymatic components including MraY (Gaballah et al., 2011).

As chlamydial MreB may serve an important function in the division of this bacterium, we sought to identify potential interaction partners by using the Bacterial Adenylate Cyclase Two Hybrid (BACTH) system as a surrogate approach to study molecular details of *Chlamydia* biology. Indeed, owing to the unusual and complex growth requirements of *Chlamydia*, there are currently no molecular tools for generating conditional depletion systems in this pathogen, and analysis of protein–protein interactions *in vivo* is problematic owing to the large amount of background host protein. The BACTH system relies on the reconstitution of adenylate cyclase activity in *E. coli* by fusing proteins of interest to two complementary fragments, T25, and T18, from the catalytic domain of the adenylate cyclase toxin of *Bordetella pertussis* (Karimova et al., 1998). The fragments are inactive when co-expressed separately, but when they are fused to proteins that interact, then the T25 and T18 fragments are brought into close proximity to allow functional complementation of enzyme activity. As the BACTH system relies on the generation of a diffusible regulatory molecule (i.e., cyclic AMP from the reconstituted cyclase activity), it allows for the separation of the protein–protein interaction and the transcriptional apparatus. The system is thus appropriate to study a wide variety of protein–protein interactions, in particular those occurring between integral membrane proteins. This technique has been extensively used to characterize protein–protein interactions *in vivo* within a variety of contexts (see Battesti and Bouveret, 2012 for review), and we recently described a Gateway® compatible version of this system (BACTH_GW_) that facilitates analysis of targeted screening assays (Ouellette et al., 2014).

Here, we identified a chlamydial homolog of RodZ/YfgA, Ct009, by performing a small-scale screen from a library of Gateway® clones to look for interaction partners of chlamydial MreB. We further demonstrated the specificity of the chlamydial MreB/RodZ association by mutating two conserved aromatic residues of RodZ and showing that this abolished their interaction. Chlamydial *rodZ* is expressed as an RB-specific gene. Chlamydial RodZ failed to complement an *E. coli rodZ* mutant likely due to decreased or defective interactions with *E. coli* MreB. We also tested the interaction of chlamydial MreB with the chlamydial homolog of MraY, an integral membrane protein that catalyzes the synthesis of the first lipid intermediate (Lipid I) of the bacterial cell wall peptidoglycan. However, at variance with a prior report that also used the BACTH assay (Gaballah et al., 2011), we found that MreB did not interact with MraY. Therefore, the association of the putative chlamydial division components with the peptidoglycan synthesis machinery remains to be conclusively established. In sum, a chlamydial RodZ homolog adds to the repertoire of rod-shape determining proteins in this coccoid bacterium.

## Materials and methods

### Cloning

A list of primers and plasmids used in this study can be viewed in Supplemental Table S1. Standard protocols were used for PCR, digestion, ligation, transformation, and plasmid preparation. *E. coli* MG1655 or *C. trachomatis* L2 genomic DNA was used as template for PCR when necessary. PCR was performed using the high-fidelity Phusion DNA polymerase (Thermo Fisher, Illkirch, France), purified with a PCR purification kit (Qiagen, Courtaboeuf, France), and, when necessary, digested with the indicated restriction enzymes (FastDigest; Thermo Fisher). Empty vectors were digested with the indicated restriction enzymes in the presence of alkaline phosphatase (FastAP; Thermo Fisher). Ligation reactions were performed using T4 DNA ligase (Thermo Fisher). Transformations were performed in chemically competent *E. coli* XL1-Blue (Stratagene [Agilent], Santa Clara, CA) cells and plated on selective antibiotics in LB agar in the presence of 0.4% glucose to repress expression. The Q5 site-directed mutagenesis kit was used, with the indicated mutagenesis primers in Supplemental Table S1, to mutate the MreB interaction residues in *rodZ* following the manufacturer's guidelines (New England Biolabs, Ipswich, MA). Medium and other chemicals were obtained from Sigma (St. Louis, MO) except where noted.

### Gateway® recombination reactions

The BP and LR (refers to the att sites) recombination reactions were performed according to the manufacturer's guidelines (Invitrogen [Life Technologies], Grand Island, NY). For all chlamydial full-length constructs except *mreB*, the indicated gene was obtained from a partial library consisting of 280 *C. trachomatis* serovar D ORFs cloned into the pDONR221 vector as constructed and sequence-verified by the Pathogen Functional Genomic Resource Center (http://pfgrc.jcvi.org). For chlamydial *mreB*, an *attB*-flanked PCR product was used in a BP reaction to insert the gene of interest into the *attP*-flanked pDONR221 to generate an *attL*-flanked ORF, which was then sequence-verified. The *attL*-flanked ORF was then recombined into the *attR*-flanked BACTH-DEST plasmids using the LR reaction to generate an *attB*-flanked ORF within the BACTH vectors (Ouellette et al., 2014). The insert was subsequently sequenced for orientation.

### Bacth and β-galactosidase assays

BACTH interactions were performed as previously described (Karimova et al., 2005) using the adenylate cyclase mutant (Δ cya) strain of *E. coli*, DHT1. Briefly, chemically competent DHT1 were co-transformed with each plasmid to be tested and plated on M63 minimal medium agar containing selective antibiotics, 40 μ g/mL X-gal (Thermo Fisher), 0.5 mM IPTG (Thermo Fisher), 0.04% casamino acids, and 0.2% maltose (Karimova et al., 1998). Plates were incubated at 30°C for up to 5 days for interactions. Only bacteria exhibiting adenylate cyclase activity are able to support robust growth on minimal medium with maltose as the sole sugar source. A positive control of the cytosolic zip constructs (Karimova et al., 1998) was used whereas negative controls included tests vs. either the empty BACTH plasmids or non-related chlamydial inner membrane proteins, Ct471 or GlnP/Ct129 (Ouellette et al., 2012). Eight colonies from each plate were cultured for 24 h at 30°C in 96-well plate format in 300 μ L minimal medium broth containing selective antibiotics, IPTG, casamino acids, and maltose. These cultures were diluted to 1 mL in minimal medium (without supplements) the following day. A blank of medium only was also included for background levels. 200 μ L was used for OD_600_ measurement and 200 μ L was used to lyse the bacteria, using chloroform and sodium dodecyl sulfate, for the β-galactosidase measurement and subsequently incubated with 0.4% ONPG for 10–20 min. The reaction was stopped by adding Na_2_CO_3_ and visualized at OD_405_. β-galactosidase activity is expressed as 1000^*^ ([OD_405_ − blank]/[OD_600_ − blank])/min. All interaction tests were performed a minimum of two times on plates with eight colonies from each plate analyzed for β-galactosidase activity.

### RT-qPCR

Assays to quantify the indicated transcripts were performed essentially as described previously (Ouellette et al., 2005, 2006). Briefly, total RNA was collected from infected cells at the indicated times using Trizol (Invitrogen) and treated with Turbo DNAfree (Ambion [Life Technologies]) to remove contaminating DNA, according to the manufacturer's guidelines. 1 μ g DNA-free RNA was reverse-transcribed with random nonamers (New England Biolabs, Ipswich, MA) using SuperScript III RT (Invitrogen) according to the manufacturer's instructions. Equal volumes of cDNA were used in qPCR reactions with SYBR Green (Quanta Biosciences, Gaithersburg, MD) and measured on an ABI 7300 system (Applied Biosystems [Life Technologies]). Duplicate DNA samples were collected from the same experiment using DNeasy Tissue kit (Qiagen). Chlamydial genomes were quantified from equal amounts of total DNA by qPCR as above and used to normalize transcript data as described (Ouellette et al., 2005, 2006).

### *E. coli* RodZ mutant and complementation

The *rodZ* mutant strain (FB60/pTB63) has been previously characterized (Bendezú et al., 2009). This strain or DH5α were transformed with plasmids pLP173 (expressing GFP-Ctr-RodZ), pLP174 (expressing GFP-Ec-RodZ), or the empty vector pMLB1113Δ H3 (*bla lacI^q^* P_lac_::-). Strains were grown overnight at 30°C in LB medium with 50 μ g/mL ampicillin and 0.2% glucose (and 5 μ g/mL tetracycline for strains harboring pTB63). Overnight cultures were diluted 1:100 in M9-maltose medium with 50 μ g/mL ampicillin and 25 μ M IPTG and grown at 30°C until mid-log phase (OD_600_ = 0.4–0.6). Samples were visualized on a Zeiss Axioplan-2 microscope and imaged at 1000× magnification as described (Johnson et al., 2002).

## Results

### Interactions of the bacterial cytoskeletal protein, MreB

**Figure 1:**
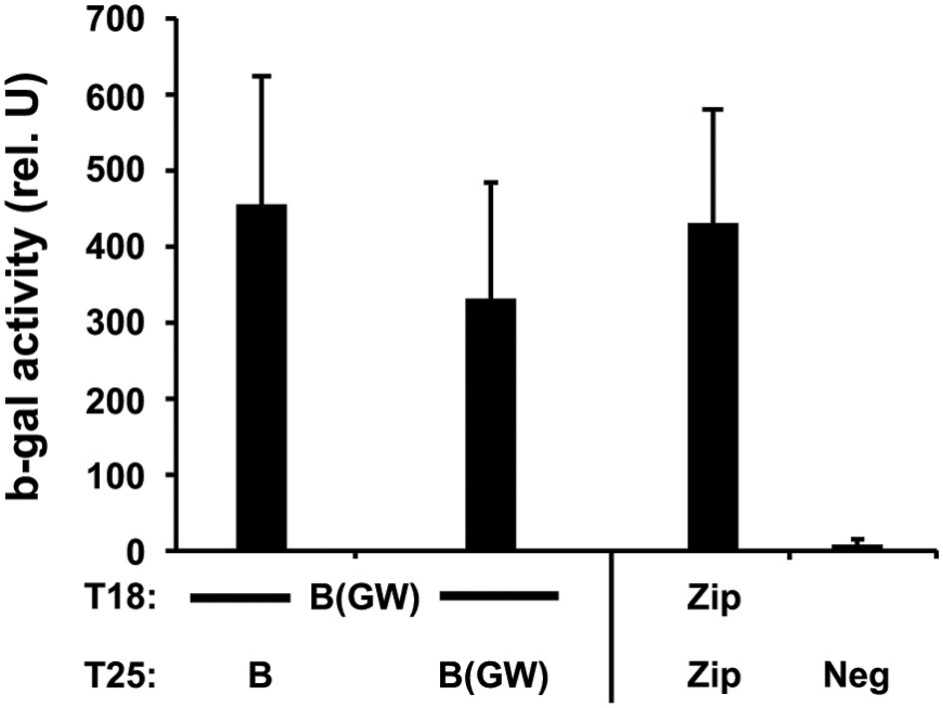
**Interactions of chlamydial MreB with itself**. Interaction assays of the indicated MreB hybrids expressed from either the Gateway® (GW) or the standard BACTH vectors. Δ cya *E. coli* DHT1 were co-transformed with the indicated constructs and plated on selective medium containing IPTG and X-gal. ß-galactosidase (ß-gal) activity was measured from eight colonies per test condition per experiment, with at least two experiments performed, after overnight culture in selective medium with IPTG and is expressed as pooled average relative units with standard deviation. A positive interaction is considered if the test interaction has 5-fold greater activity compared to negative controls. The positive control of T25-Zip and T18-Zip was used. Neg = negative control consisting of pooled data from tests of constructs vs. empty vectors or unrelated chlamydial inner membrane proteins, Ct471 or GlnP/Ct129.

MreB is a bacterial homolog of actin and is thought to serve an essential role in maintaining the rod-shape of bacillus bacteria. MreB can form filaments and use ATP, like actin (van den Ent et al., 2001). To characterize the interacting properties of chlamydial MreB with the BACTH system, we cloned the full-length *mreB* either into the Gateway® compatible (BACTH_GW_) vectors or into the original BACTH vectors (although we could not obtain a correct insertion of MreB into the high-copy pUT18C vector). Two-hybrid assays revealed that each of the chlamydial MreB T25 clones, expressed from *pKT25-mreB* or *pST25-mreB(GW)*, was capable of interacting with the T18 MreB Gateway® clone, expressed from *pUT18C-mreB(GW)* (Figure 1), thus confirming the ability of chlamydial MreB to oligomerize.

Given the proposed role of chlamydial MreB as a functional substitute for FtsZ (Ouellette et al., 2012) and the paucity of annotated cell division proteins in this bacterium, we wanted to identify interaction partners of MreB since such proteins might function in cell division. To this end, we used our Gateway® library of chlamydial ORFs to perform a small-scale biased screen using chlamydial MreB as bait. Table 1 summarizes the results. Amongst the 21 genes we analyzed, we identified only three positive interactions, two of which were previously described and included as positive controls (Ouellette et al., 2012): MreB (Ct709), FtsK (Ct739), and Ct009. However, we did not detect an interaction with MraY or MurG, at variance with a previous report from Gaballah et al. (2011).

**Table 1:** **Small-scale BACTH screen to identify proteins that interact with chlamydial MreB**.

**Gene ID**^1^****	**Annotation**	**Interaction**
Ct005	Hypothetical	−
Ct009	*yfgA*	+
Ct012	*ybbP*	−
Ct129^−^	*glnP*	−
Ct144	Hypothetical	−
Ct174	Hypothetical	−
Ct270^−^	*ftsI/pbp3*	−
Ct277	Hypothetical	−
Ct303	Hypothetical	−
Ct357	Hypothetical	−
Ct446	*euo*	−
Ct471	Hypothetical	−
Ct482	Hypothetical	−
Ct605	Hypothetical	−
Ct682^−^	*pbp2*	−
Ct709^+^	*mreB*	+
Ct726	*rodA*	−
Ct739^+^	*ftsK*	+
Ct756	*murF*	−
Ct757^#^	*mraY*	−
Ct760	*ftsW*	−
Ct761^#^	*murG*	−

1 Using the numbering scheme of Stephens et al. (1998) at http://stdgen.northwestern.edu.

+ Included as positive or

− negative control (Ouellette et al., 2012).

# Reported as BACTH interacting partner by Gaballah et al. (2011).

**Figure 2:**
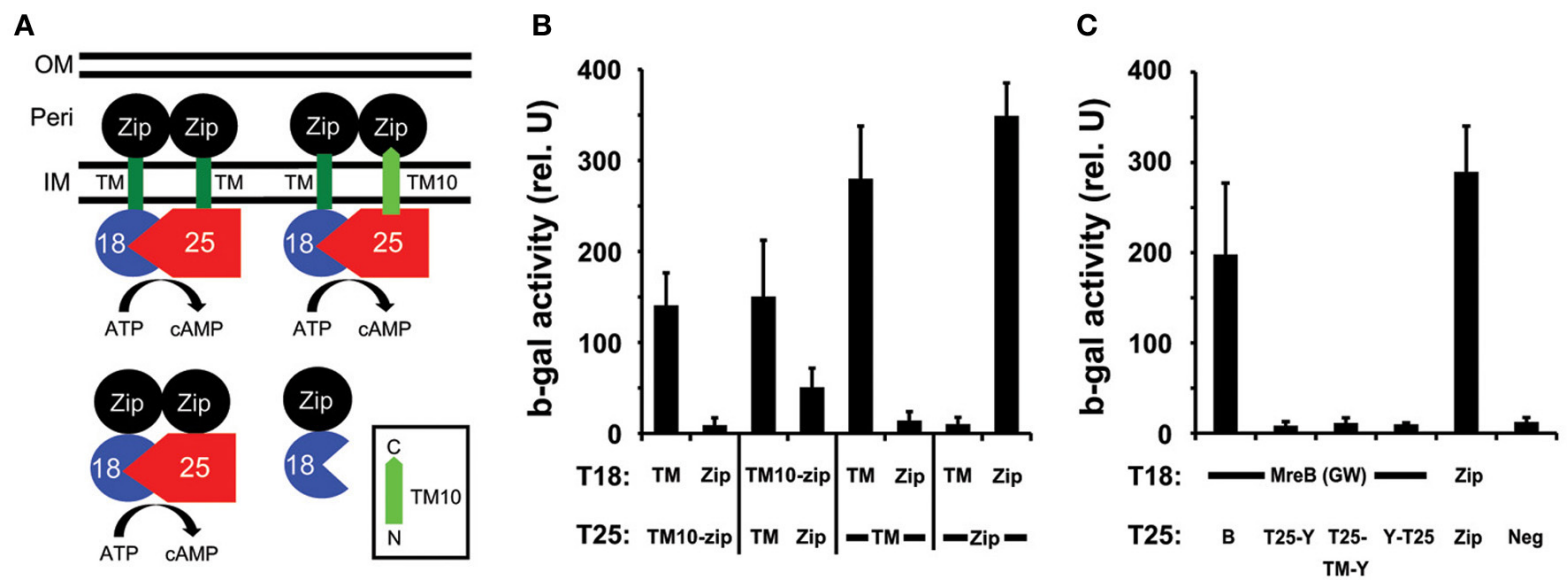
**Determination of the orientation of MraY and its interaction with MreB using the BACTH system. (A)** The 26 amino acids encoding TM10 of MraY (residues 311–336; light green with predicted orientation boxed in bottom right of **A**) were cloned between the leucine zipper domain, Zip (black circle), and the T18 (blue pie shape) or T25 (red pentagon) fragment of adenylate cyclase to create the product T25-TM10-zip or T18-TM10-zip. These were tested against the positive control vectors encoding T25-Zip or T18-Zip and T25-TM-zip or T18-TM-zip (indicated as TM in **(B,C)** with the transmembrane domain of OppB from *E. coli*; dark green). The diagram illustrates the expected interactions with T18-TM-zip if T25-TM10-zip has its C-terminus in the periplasm as predicted. Note that the Zip domains must bring together the T18 and T25 fragments to detect an interaction. IM, inner membrane; Peri, Periplasm; OM, outer membrane. **(B)** Quantification of the ß-galactosidase activity from the BACTH tests of TM10 from MraY as described in **(A)**. **(C)** Chlamydial MreB does not interact with MraY. T18-MreB(GW) was tested against T25-MreB(GW) [B] or T25-MraY [T25-Y], T25-TM-MraY expressed from the BACTH-TM plasmid [T25-TM-Y], or MraY-T25 [Y-T25]. Note that only the T25-TM-MraY fusion respects the native topology of MraY as an extra TM is inserted between the cytosolic T25 domain and the N-terminal extremity of MraY. ß-galactosidase assays were performed as described in the legend to Figure 1.

To verify the predicted topology of chlamydial MraY, we took advantage of recent work showing that the charge characteristics at the C-terminus of the last transmembrane (TM) domain of a polytopic membrane protein determine its orientation in the membrane (Seppala et al., 2010). We thus inserted the C-terminal 26 amino acids encoding the last TM domain (TM10) of chlamydial MraY, as predicted by TOPCONS (Supplemental Figure S1; Bernsel et al., 2009), between the T25 or T18 fragment and the leucine zipper (Zip) domain as described (Figure 2A; Ouellette et al., 2014). We then tested with the BACTH system whether these fusion proteins (T25-TM10-zip and T18-TM10-zip) interacted with either a cytosolic Zip (e.g., T25-Zip) or a periplasmic zip fused to the T25 domain via the first TM domain of *E. coli* OppB (e.g., T25-TM-zip; Ouellette et al., 2014). As shown in Figure 2B, the T25-TM10-zip interacted specifically with the T18-TM-zip but not T18-Zip, and, conversely, the T18-TM10-zip interacted strongly with the T25-TM-zip and to a much lower extent with the T25-Zip. This indicates that the chlamydial MraY TM10 segment is indeed driving the zip motif to the periplasm. Therefore, *Chlamydia* MraY encodes its C-terminus, and by extension, its N-terminus, in the periplasm in accordance with the topology of *E. coli* MraY (Bouhss et al., 1999).

We next re-examined whether chlamydial MreB was capable of interacting with MraY using the BACTH_GW_ system with (and without) the additional OppB TM to favor the native topology of MraY (Ouellette et al., 2014). The chlamydial *mraY* gene was recombined into all BACTH_GW_ vectors: pST25-DEST, pUT18C-DEST, pSNT25-DEST, pSTM25-DEST, and pUTM18C-DEST. We then transformed the Δ *cya E. coli* with different pair-wise combinations of the MreB and MraY constructs and assayed the β-galactosidase activity to quantify the interactions between these different hybrid proteins. We did not detect an interaction between MreB and MraY when MraY was expressed from any BACTH_GW_ vector (Figure 2C and data not shown) and neither did we detect a specific interaction of *E. coli* MreB with its MraY (data not shown). Therefore, these BACTH analyses do not support an association between MreB and MraY.

### Identification of a chlamydial RodZ homolog

**Figure 3:**
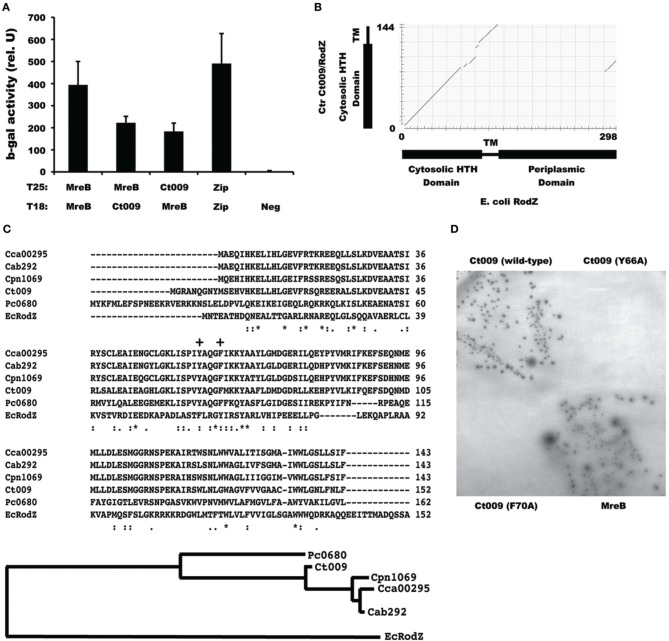
**Identification of a chlamydial *rodZ* homolog. (A)** Interaction of chlamydial MreB with Ct009 in the BACTH_GW_ system. A positive control of T25-Zip vs. T18-Zip is shown. The ß-galactosidase assays and negative controls are as described for Figure 1. **(B)** Alignment of chlamydial Ct009/RodZ (y-axis) with *E. coli* RodZ (x-axis) with corresponding domains schematically represented. Note that the chlamydial RodZ homolog is missing the periplasmic domain. **(C)** ClustalW sequence alignment and phylogenetic relatedness of chlamydial Ct009 homologs, with corresponding gene numbering, vs. amino acids 1–152 of *E. coli* RodZ with conserved residues indicated by [^*^] and related residues by dots (e.g., [:]). The “+” indicates the MreB-interaction residues as described by van den Ent et al. (2010). Cca, *C. caviae*; Cab, *C. abortus*; Cpn, *C. pneumoniae*; Ct, *C. trachomatis*; Pc, *Protochlamydia ameobophila*; Ec, *E. coli* **(D)** BACTH test of T25-MreB vs. the indicated T18 fusion proteins. Only positive interactions lead to growth on minimal medium with maltose with corresponding production of ß-galactosidase.

We detected, and subsequently validated, from our initial screen an interaction with the chlamydial protein Ct009, annotated as YfgA (Stephens et al., 1998; Figure 3A). An alignment of Ct009 against *E. coli* RodZ showed that Ct009 aligns quite well with RodZ in spite of the low level of identity between the proteins (25% identical/40% similar over 43% of *E. coli* RodZ; E value = 6e-07; Figure 3B). Interestingly, *Chlamydia* RodZ lacks the large periplasmic domain that was shown to be dispensable for cell shape maintenance in *E. coli* (Bendezú et al., 2009). Ct009 homologs are present in all chlamydial genomes (Figure 3C and data not shown), an important consideration for a potential cell division component. Within the *Chlamydiales*, the *Protochlamydia* RodZ homolog grouped outside the other chlamydial species whereas the animal species clustered with each other and were separate from *C. trachomatis* (Figure 3C).

In *E. coli*, two aromatic residues (F60 and Y64) within the HTH domain have been shown to be critical for its interaction with MreB (van den Ent et al., 2010). To further validate the specific interaction of Ct009 with MreB, we mutated the corresponding residues (Y66 and F70) within the HTH domain of Ct009 (indicated by + in Figure 3C). We then tested these variants (Y66A and F70A) against MreB in the BACTH assay. As shown in Figure 3D, we did not detect any interaction between these Ct009 variants and MreB, demonstrating the specificity of the Ct009/MreB association. In sum, these data strongly support the conclusion that Ct009 is a chlamydial RodZ homolog.

### *ct009/rodZ* is transcribed as an RB-specific gene

**Figure 4:**
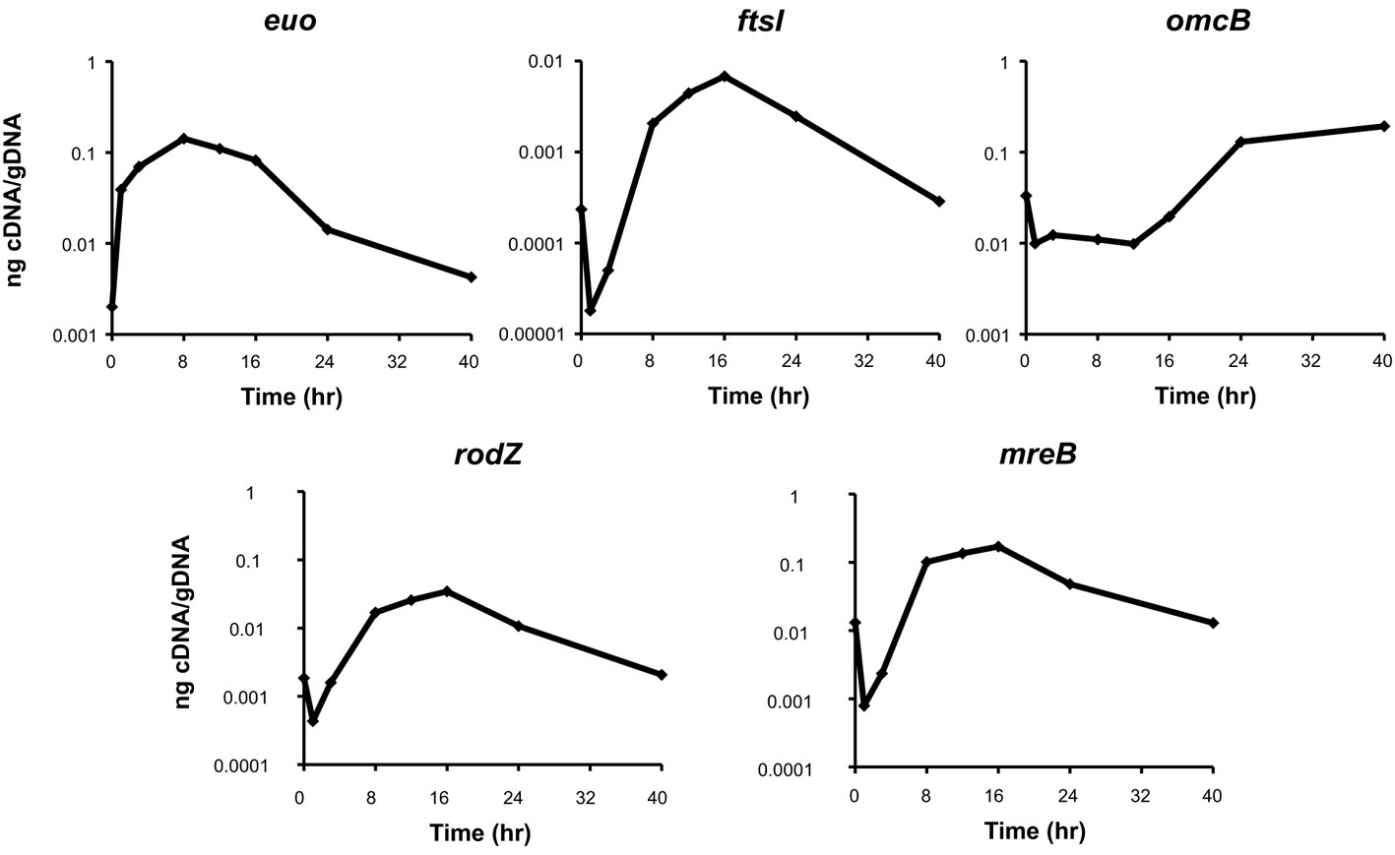
**Transcriptional analysis of *ct009*/*rodZ* during the chlamydial developmental cycle**. The indicated genes were analyzed by RT-qPCR over a time course of infection with *C. trachomatis*. The top panels show control genes for EB-to-RB transition (*euo*), RB growth and division (*ftsI*), and RB-to-EB transition (*omcB*). Transcription of *rodZ* and *mreB* is shown in the bottom panels and exhibits an *ftsI-*like expression pattern indicative of RB growth and division. DNase-treated total RNA was reverse transcribed and analyzed by qPCR. Data were normalized to genome numbers and are representative of two biological replicates analyzed in triplicate. Standard deviations were generally less than 5% of the average.

As a first step to determining a putative function for Ct009/RodZ, we measured its transcription during the chlamydial developmental cycle to assign it a temporal pattern of expression. RNA was collected at various time points to reflect the differentiation of EB to RB (i.e., early cycle), RB growth and division (i.e., mid cycle), and re-differentiation from RB to EB (i.e., late cycle) (Shaw et al., 2000). A panel of representative genes of these temporal classes was examined as a control with *euo* representing early (Wichlan and Hatch, 1993), *ftsI* (a *bona fide* cell division gene) representing mid, and *omcB* representing late cycle stages (Everett and Hatch, 1991). As can be seen in Figure 4, *ct009/rodZ* transcription most closely resembles the mid cycle transcriptional pattern of *ftsI* and markedly differs from the expression pattern of genes specific to the EB to RB (*euo*) or RB to EB (*omcB*) transition. Furthermore, the pattern of *ct009/rodZ* expression closely resembled that of *mreB*, its binding partner. Therefore, we conclude that, like MreB, Ct009/RodZ functions as an RB-specific product and most likely as a cell division-related protein.

### Ct009/RodZ localizes to membranes in *E. coli* but fails to complement an *E. coli* RodZ mutant

**Figure 5:**
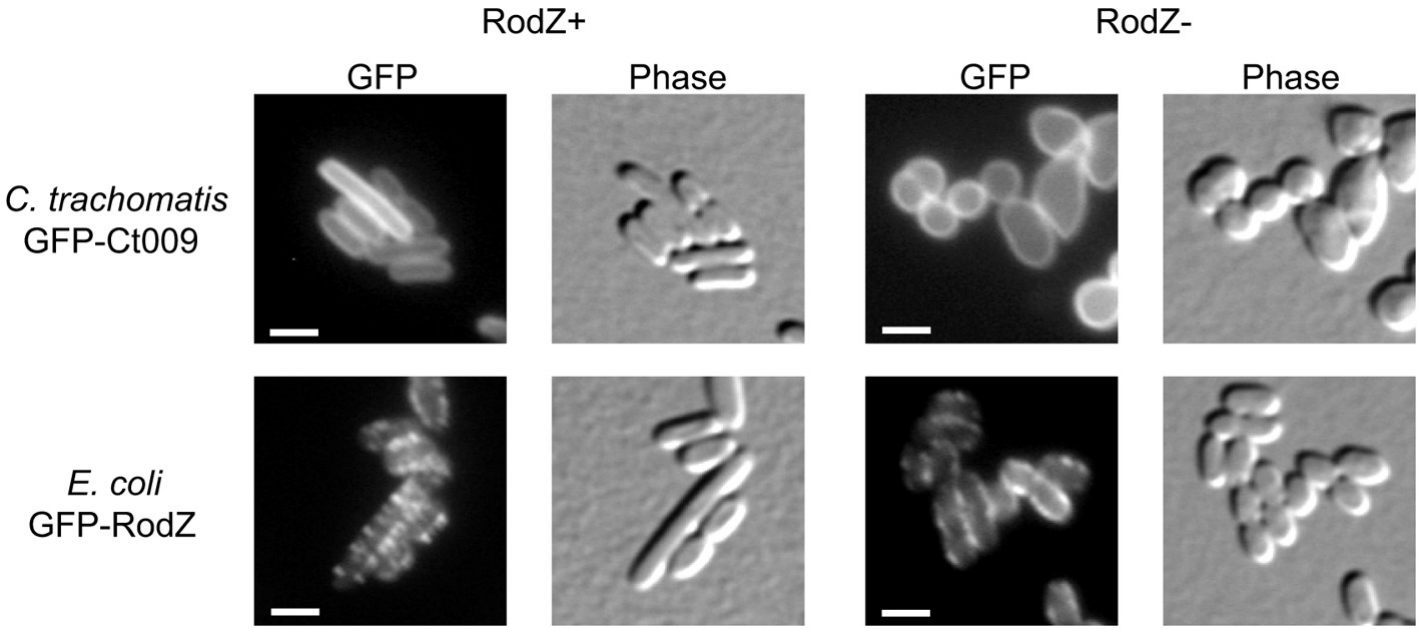
**Test of complementation of Ct009/RodZ in *E. coli***. An *E. coli* wild-type strain (RodZ+) or the Δ rodZ FB60/pTB63 mutant *E. coli* strain (RodZ−) was transformed with the indicated GFP constructs. Expression was induced and the localization of the RodZ protein and the cell shape were monitored by fluorescence microscopy. The absence of RodZ leads to loss of the rod shape with resulting irregular coccoid morphology. Scale bar = 2 μm.

Since division proteins are essential for viability, putative candidate division components are typically investigated using conditional depletion systems, temperature-sensitive mutants, or by over-expressing key proteins of the division apparatus (e.g., FtsZ) to compensate for the lack of the candidate. These experimental approaches do not yet exist for *Chlamydia*. Thus, to further explore the function of Ct009 as a RodZ homolog, we used a complementing approach in a surrogate *E. coli* RodZ mutant strain (FB60/pTB63) where *rodZ* has been deleted, but the cells are kept viable by the presence of a plasmid encoding *ftsQAZ* (Bendezú et al., 2009). In this RodZ^−^ background, the bacteria lose their rod-shape morphology and are round or irregularly shaped but continue to divide. GFP-Ct009 or GFP-RodZ (from *E. coli*) was expressed in either a RodZ wild-type background or the Δ rodZ mutant strain. In wild-type cells, GFP-Ct009 localized uniformly to the membranes whereas GFP-RodZ from *E. coli* adopted the characteristic spotty helical-like membrane localization pattern consistent with previous observations (Figure 5; Bendezú et al., 2009). In the Δ rodZ cells, GFP-Ct009 showed the same membrane localization pattern as in wild-type cells, and it failed to correct their shape defects (Figure 5). Conversely, GFP-RodZ from *E. coli* was able to do so and again showed its characteristic localization pattern. The empty vector control showed no effect on the Δ rodZ mutant strain, as expected (Supplemental Figure S2). Thus, Ct009 correctly localizes to the membrane in *E. coli* but is unable to complement the shape defect of *E. coli* cells that lack native RodZ.

### Failure of Ct009/RodZ to complement may be due to its inability to recapitulate essential interactions

**Figure 6:**
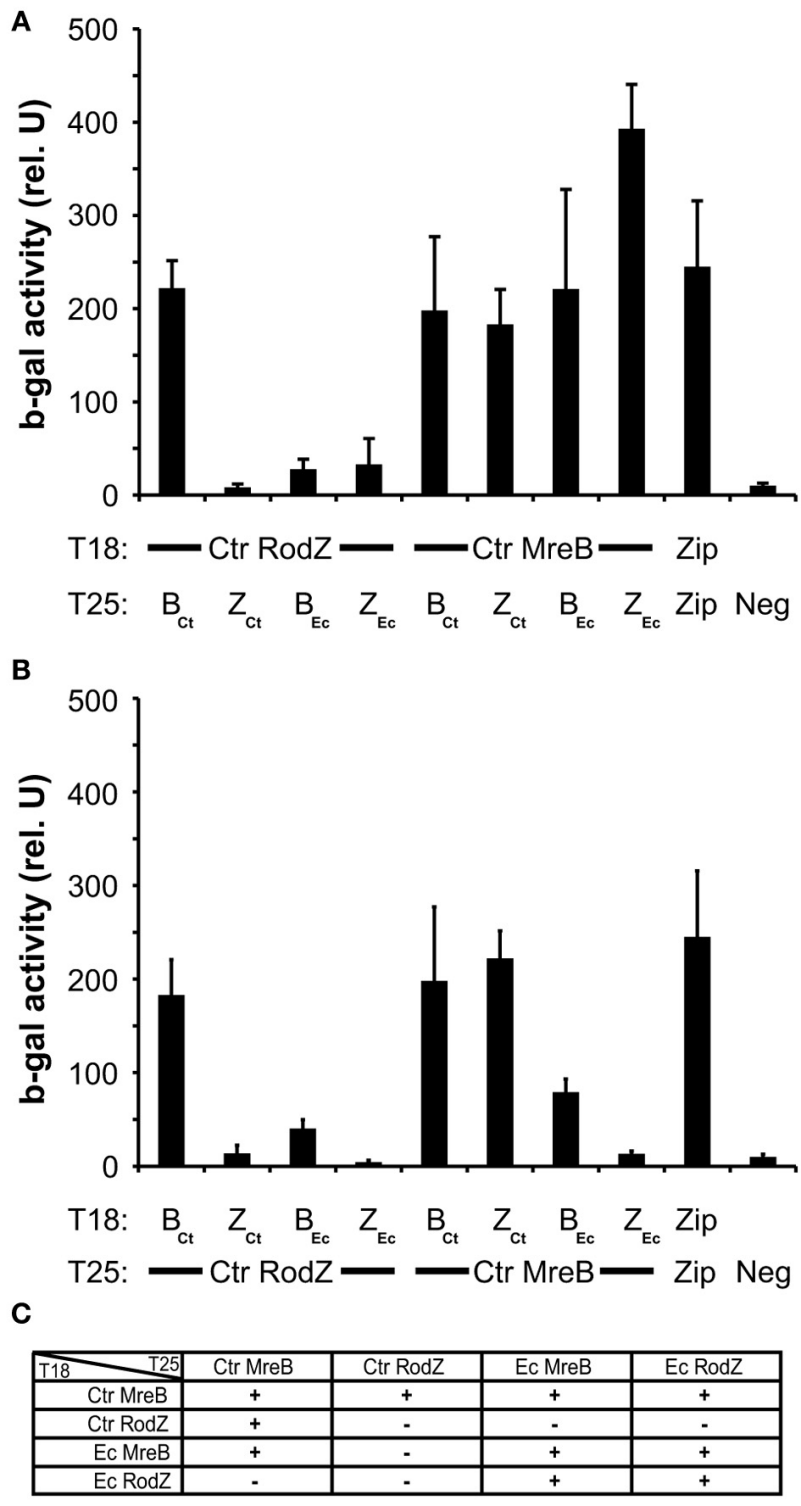
**Heterologous interactions between MreB and RodZ from *Chlamydia* and *E. coli***. The indicated constructs from the two species were tested in pair-wise fashion for their ability to interact in the BACTH assay. **(A,B)** Quantification of the ß-galactosidase activity as described in the legend of Figure 1. Ctr, *Chlamydia trachomatis*; Ec, *Escherichia coli*; B, MreB; Z, RodZ **(C)** Summary of heterologous interactions.

To explore in further detail why chlamydial RodZ failed to complement the *E. coli* depletion mutant, we tested heterologous interactions between the chlamydial and *E. coli* RodZ and MreB proteins. Pair-wise BACTH interaction tests were performed between these proteins in each orientation and combination (Figure 6). Chlamydial MreB interacted robustly with chlamydial RodZ in all combinations. Chlamydial MreB also interacted efficiently with *E. coli* MreB, perhaps not surprising given the high level of conservation (57% identical/71% similar over 95% of *E. coli* MreB; E value = 1e-125). Further, chlamydial MreB interacted with *E. coli* RodZ but only when the latter was encoded on the low copy vector (i.e., with T25-RodZ). Conversely, chlamydial RodZ failed to show an interaction with *E. coli* MreB or RodZ and was not capable of dimerizing as was shown for other RodZ homologs (Figure 6, Supplemental Figure S3; White et al., 2010). We conclude that the lack of complementation of the *E. coli* Δ rodZ mutant by Ct009 is most likely due to its inability to recapitulate essential interactions with the *E. coli* shape determining protein MreB.

## Discussion

*Chlamydia* is an important human pathogen that presents certain challenges with which to work. A complicating factor for performing interaction or localization studies is the relatively small size of the organism and its obligate dependence on a host cell for growth. Characterization of interacting properties of chlamydial proteins is one approach to gain further insight about the unique biology of this pathogen. Therefore, we used a bacterial two-hybrid system, the BACTH_GW_, to examine chlamydial protein–protein interactions, with a particular emphasis on the study of cell division proteins. Intriguingly, *Chlamydia* is remarkable in lacking the highly conserved and essential FtsZ protein that plays a key role in organizing the bacterial divisome (Begg and Donachie, 1985). We proposed recently that the cytosolic protein MreB may serve a similar role as a functional substitute for FtsZ in chlamydial cell division (Ouellette et al., 2012). Here, we searched for additional chlamydial partners of MreB, hypothesizing that they might be putative cell division proteins in this bacterium. We found that Ct009, a membrane protein with homology to RodZ/YfgA, efficiently interacted with chlamydial MreB. We are currently pursuing further BACTH_GW_ screens to identify additional components of the chlamydial cell division apparatus.

The identification of a RodZ homolog in *Chlamydia* adds to the repertoire of rod-shape determining proteins in this coccoid bacterium. *Chlamydia* has undergone extensive genome reduction in adapting to its obligate intracellular life style. Every open reading frame is transcriptionally expressed during the developmental cycle (Belland et al., 2003), and there are few pseudogenes present in chlamydial genomes (Stephens et al., 1998). Therefore, the majority of genes are likely essential. Consequently, the presence of rod-shape determining genes in *Chlamydia* should be interpreted as necessary for its survival. In bacilli, the rod-shape determining proteins direct the peptidoglycan machinery to the cell wall to allow for side-wall growth whereas a different set of machinery is used to generate a cell wall (i.e., septum) in the division plane (van der Ploeg et al., 2013). *Chlamydia* lacks many of the canonical cell division proteins including FtsZ, thus we have hypothesized that it has co-opted the rod-shape determining proteins to function in cell division (Ouellette et al., 2012). Interestingly, although *Chlamydia* encodes a RodZ homolog, Ct009, this protein was unable to complement an *E. coli* RodZ mutant in spite of its localization to membranes. Given the inability of chlamydial RodZ to re-capitulate the essential interaction with *E. coli* MreB, this finding is perhaps not surprising. Further, given the coccoid shape of *Chlamydia*, it stands to reason that the chlamydial RodZ may have lost essential determinants that govern the rod-shape and/or gained other determinants that support a different function in *Chlamydia*. We are currently exploring this.

Very recently, Jacquier et al. (2014) have shown that a *Waddlia* homolog of Ct009 localizes to apparent division sites in this bacterium. Given our interaction data showing that MreB interacts with Ct009/RodZ, these data combine to show a likely role of chlamydial RodZ in cell division. Based on combinatorial antibiotic treatments as we have previously used (Ouellette et al., 2012), Jacquier et al. (2014) also suggested that MreB functions downstream from peptidoglycan precursors in the division process. Part of their rationale for this conclusion is based on the supposed interaction of MreB with MraY and MurG, both of which are involved in peptidoglycan synthesis, as reported by Gaballah et al. (2011). These authors also used the BACTH assay to suggest that MraY and MurG could bind to MreB in *Chlamydia pneumoniae*. However, the BACTH interaction assays of Gaballah et al. (2011) yielded only very low levels of β-galactosidase activity (less than twice the negative control levels whereas a positive interaction should have 5-fold greater activity). Thus, the interpretation of MreB's interacting with MraY and MurG from these data is flawed. One major caveat with the MraY study is that MraY is predicted to have both its N- and C-termini in the periplasm (Bouhss et al., 1999; Chung et al., 2013). Thus, direct fusion of the T25 or T18 cyclase fragments to MraY, as done by Gaballah et al. (2011), is expected to result in a non-native insertion of the protein into the membrane, or alternatively, in the targeting of the T25 or T18 fragment to the periplasm where they would not be functional. We did not detect interactions between MreB and MraY or MurG using the same experimental approach as Gaballah et al. (2011) and argue that the interpretation of their data is incorrect. Consequently, any conclusions based on their data should be carefully interpreted, and further experimental work will be required to establish such an interaction.

In sum, we have shown the utility of examining protein–protein interactions to identify components of pathways in *Chlamydia* that were previously not annotated. Without being able to implement conditional knockout or depletion systems, this approach will aid in our understanding of chlamydial microbiology. Indeed, for proteins that are essential in *Chlamydia*, this may be the preferred approach.

## Author contributions

Scot P. Ouellette designed and performed experiments, analyzed data, and wrote the manuscript. Kelsey J. Rueden, Emilie Gauliard, and Logan Persons performed experiments. Piet A. de Boer and Daniel Ladant designed experiments, analyzed data, and wrote the manuscript.

### Conflict of interest statement

The authors declare that the research was conducted in the absence of any commercial or financial relationships that could be construed as a potential conflict of interest.

## Supplementary material

Supplemental Figure S1 **TOPCONS membrane topological analysis of chlamydial MraY (Ct757)**. Note the presence of 10 TM domains with both the N- and C-termini located “outside.”

Supplemental Figure S2 **The empty vector pMLB1113Δ H3 has no effect on the cell shape of the Δ *rodZ* mutant strain, FB60/pTB63**. Cells from the transformed strain were imaged under phase contrast.

Supplemental Figure S3 **BACTH interactions between *E. coli* MreB and RodZ**. Quantification of the ß-galactosidase activity as described in the legend of Figure 1.

Supplemental Table S1 **List of primers, plasmids, and strains used in the study**.
