# Supplementary material for: Analysis of MreB interactors in Chlamydia reveals a RodZ homolog but fails to detect an interaction with MraY
Source: Front Microbiol. 2014 Jun 6;5:279. doi: 10.3389/fmicb.2014.00279 (PMC4047632; doi:10.3389/fmicb.2014.00279)
Supplement: Supplemental Figure S1 — TOPCONS membrane topological analysis of chlamydial MraY (Ct757). Note the presence of 10 TM domains with both the N- and C-termini located “outside.” [file Presentation1.ZIP › Supp Table.DOCX]

Supplemental Table 1. *E.coli* strains and plasmids

| Strain | Relevant genotype ^a^ | | Source or Reference |
| --- | --- | --- | --- |
| DH5α | *hsdR*17 *deoR* *recA*1 *endA*1 *phoA supE*44 *thi*-1 *gyrA*96 *relA*1  Δ(*lacZYA-argF*)U169 φ80d*lacZ*ΔM15 | | Gibco BRL (Life Technologies) |
| DHT1  FB60* | F^-^ *glnV44* (AS) *recA1 endA1 gyrA96* (Nal^R^) *thi-1 hsdR17 spoT1 rfbD1*  *cya-854 ilv-691* ::Tn*10* (Tet^R^)  TB28, *rodZ<>aph* | | (1)  ([2](#_ENREF_1)) |
| TB28 | *ilvG* *rfb*50 *rph*1 *lacIZYA*<>*frt* | | ([4](#_ENREF_3)) |
| XL1-Blue | *recA1 endA1 gyrA96* (Nal^R^) *thi-1 hsdR17 supE44 relA1 lac*  [F’ *proAB lacI^q^ZΔM15* Tn*10* (Tet^R^)] | | Stratagene (Agilent) |
|  |  | |  |
| Construct | Relevant genotype ^a^ | ori | Source or Reference |
| Plasmids: |  |  |  |
| pDONR221  pKT25  pUT18C  pKT25-zip  pUT18C-zip  pKTM25-zip  pUTM18C-zip  pKT25-TM10-zip  pUT18C-TM10-zip  pST25-DEST  pSNT25-DEST  pUT18C-DEST  pSTM25-DEST  pUTM18C-DEST  pENTR005  pENTR009  pENTR012  pENTR174  pENTR270  pENTR277  pENTR303  pENTR357  pENTR446  pENTR482  pENTR682  pENTR709  pENTR726  pENTR739  pENTR756  pENTR757  pENTR760  pST005  pST009  pST012  pST174  pST270  pST277  pST303  pST357  pST446  pST482  pST605  pST682  pST709  pST726  pST739  pST756  pST757  pST760  pSNT757  pSTM757  pKT129  pKT144  pKT471  pKT709  pUT009  pUT709  pUT757  pUTM757  pCH371 | *aph* *att*P1-[*cat ccdB*]-*att*P2  *aph* P_lac_::*t25*  *bla* P_lac_::*t18*  *aph* P_lac_::*t25-zip*  *bla* P_lac_::*t18-zip*  *aph* P_lac_::*t25-TM-zip*  *bla* P_lac_::*t18-TM-zip*  *aph* P_lac_::*t25-TM10_mraY_-zip*  *bla* P_lac_::*t18-TM10_mraY_-zip*  *aadA* P_lac_::*t25-att*R1-[*cat ccdB*]-*att*R2  *aadA* P_lac_::*att*R1-[*cat ccdB*]-*att*R2-*t25*  *bla* P_lac_::*t18-att*R1-[*cat ccdB*]-*att*R2  *aadA* P_lac_::*t25-TM-att*R1-[*cat ccdB*]-*att*R2  *bla* P_lac_::*t18-TM-att*R1-[*cat ccdB*]-*att*R2  *aph* *att*L1-[*ct005*]-*att*L2  *aph* *att*L1-[*rodZ*]-*att*L2  *aph* *att*L1-[*ybbP*]-*att*L2  *aph* *att*L1-[*ct174*]-*att*L2  *aph* *att*L1-[*ftsI*]-*att*L2  *aph* *att*L1-[*ct277*]-*att*L2  *aph* *att*L1-[*ct303*]-*att*L2  *aph* *att*L1-[*ct357*]-*att*L2  *aph* *att*L1-[*euo*]-*att*L2  *aph* *att*L1-[*ct482*]-*att*L2  *aph* *att*L1-[*pbp2*]-*att*L2  *aph* *att*L1-[*mreB*]-*att*L2  *aph* *att*L1-[*rodA*]-*att*L2  *aph* *att*L1-[*ftsK*]-*att*L2  *aph* *att*L1-[*murF*]-*att*L2  *aph* *att*L1-[*mraY*]-*att*L2  *aph* *att*L1-[*ftsW*]-*att*L2  *aadA* P_lac_::*t25-att*B1-[*ct005*]-*att*B2  *aadA* P_lac_::*t25-att*B1-[*rodZ*]-*att*B2  *aadA* P_lac_::*t25-att*B1-[*ybbP*]-*att*B2  *aadA* P_lac_::*t25-att*B1-[*ct174*]-*att*B2  *aadA* P_lac_::*t25-att*B1-[*ftsI*]-*att*B2  *aadA* P_lac_::*t25-att*B1-[*ct277*]-*att*B2  *aadA* P_lac_::*t25-att*B1-[*ct303*]-*att*B2  *aadA* P_lac_::*t25-att*B1-[*ct357*]-*att*B2  *aadA* P_lac_::*t25-att*B1-[*euo*]-*att*B2  *aadA* P_lac_::*t25-att*B1-[*ct482*]-*att*B2  *aadA* P_lac_::*t25-att*B1-[*ct605*]-*att*B2  *aadA* P_lac_::*t25-att*B1-[*pbp2*]-*att*B2  *aadA* P_lac_::*t25-att*B1-[*mreB*]-*att*B2  *aadA* P_lac_::*t25-att*B1-[*rodA*]-*att*B2  *aadA* P_lac_::*t25-att*B1-[*ftsK*]-*att*B2  *aadA* P_lac_::*t25-att*B1-[*murF*]-*att*B2  *aadA* P_lac_::*t25-att*B1-[*mraY*]-*att*B2  *aadA* P_lac_::*t25-att*B1-[*ftsW*]-*att*B2  *aadA* P_lac_::*att*B1-[*mraY*]-*att*B2-*t25*  *aadA* P_lac_::*t25-TM-att*B1-[*mraY*]-*att*B2  *aph* P_lac_::*t25-glnP*  *aph* P_lac_::*t25-ct144*  *aph* P_lac_::*t25-ct471*  *aph* P_lac_::*t25-mreB*  *bla* P_lac_::*t18-att*B1-[*rodZ*]-*att*B2  *bla* P_lac_::*t18-att*B1-[*mreB*]-*att*B2  *bla* P_lac_::*t18-att*B1-[*mraY*]-*att*B2  *bla* P_lac_::*t18-TM-att*B1-[*mraY*]-*att*B2  *bla* *lacI*^q^ P_lac_::*t18-rodZ* | ColE1  pACYC  ColE1  pACYC  ColE1  pACYC  ColE1  pACYC  ColE1  pACYC  pACYC  ColE1  pACYC  ColE1  ColE1  ColE1  ColE1  ColE1  ColE1  ColE1  ColE1  ColE1  ColE1  ColE1  ColE1  ColE1  ColE1  ColE1  ColE1  ColE1  ColE1  pACYC  pACYC  pACYC pACYC pACYC pACYC pACYC pACYC pACYC pACYC pACYC pACYC pACYC pACYC pACYC pACYC pACYC pACYC pACYC pACYC pACYC pACYC  pACYC  pACYC  ColE1  ColE1  ColE1  ColE1  ColE1 | Invitrogen (Life Technologies)  (9)  (9)  (9)  (9)  (7)  (7)  (7)  (7)  (7)  (7)  (7)  (7)  (7)  PFGRC  PFGRC  PFGRC  PFGRC  (8)  PFGRC  PFGRC  PFGRC  PFGRC  PFGRC  PFGRC  (8)  PFGRC  PFGRC  This work  PFGRC  PFGRC  This work  This work  This work  This work  (8)  This work  This work  This work  This work  This work  This work  (8)  (8)  This work  (8)  This work  This work  This work  This work  This work  (8)  This work  (7)  (8)  This work  (8)  This work  This work  ([2](#_ENREF_1)) |
| pCH375  pCH356  pCH358  pLP14 | *aph* P_lac_::*mreB’-t25-‘mreB*  *bla lacI*^q^ P_lac_::*mreB’-t18-‘mreB*  *aph* P_lac_::*t25-rodZ*  *bla* *lacI*^q^ P_lac_::*gfp-t-ponA* | pACYC  ColE1  pACYC  ColE1 | (2)  (2)  (2)  ([6](#_ENREF_5)) |
| pLP173 | *bla* *lacI*^q^ P_lac_::*gfp-t*-CT*rodZ* | ColE1 | This work |
| pLP174 | *bla* *lacI*^q^ P_lac_::*gfp-t*-*rodZ* | ColE1 | This work |
| pMLB1113ΔH | *bla* *lacI*^q^ P_lac_:: | ColE1 | ([5](#_ENREF_4)) |
| pSO1 | *cat* *araC* P_BAD_::CT*rodZ* | pACYC | This work |
| pTB63 | *tet ftsQ ftsA ftsZ* | pSC101 | ([3](#_ENREF_2)) |
|  |  |  |  |

^a^Genotypes indicate when constructs encode in-frame Gfpmut2 (*gfp*), T7.tag (*t*), the CyaA T25- or T18-domain (*t25* or *t18*), and/or the first TM domain of OppB from *E. coli* (TM; see ref. 7). The *att* recombination sites for the Gateway vectors are indicated in their position relative to the gene of interest. <> denotes DNA replacement by λ red recombineering. Note that strains marked with * required an appropriate plasmid, phage, inducer, and/or medium for survival. PFGRC = Pathogen Functional Genomic Resource Center.

Constructs:

The BACTH (9) and the BACTH Gateway empty vectors (7) have been described previously.

To insert the TM10 domain of chlamydial MraY in frame into the T25-Zip or T18-Zip expressing plasmids, the following primers were used to amplify the corresponding genomic region from *C. trachomatis*: 5’-ATAGTCTGCAGT**G**AAAAGGCTGTAGTGAGGAATTTC-3’ for pUT18C-zip, 5’-ATATTCTGCAGGT**G**AAAAGGCTGTAGTGAGGAATTTC-3’ for pKT25-zip (*Pst*I sites underlined for both), and 5’- ATATAGGATCCCC**A**TCCACAAATACTGCAATGATCCC-3’ (*Bam*HI site underlined) for both. The resulting PCR product was digested and inserted into the corresponding *Pst*I and *Bam*HI sites of pKT25-zip or pUT18C-zip to create pKT25-TM10_mraY_-zip and pUT18C-TM10_mraY_-zip.

Plasmids pCH356 (2), pCH358 (2), pCH371 (2), pCH375 ([2](#_ENREF_1)), pKT709 (8), pLP14 ([6](#_ENREF_5)), and pMLB1113ΔH ([5](#_ENREF_4)) were described before.

For pENTR756, *ct756* was amplified, with flanking *att*B sites, from *C. trachomatis* genomic DNA with primers 5’- AATTAACAAGTTTGTACAAAAAAGCAGGCTTT**A**TGCGCCCTATTTTGTTGGAAGAATGG-3’ (start site is bolded) and 5’- AATTACCACTTTGTACAAGAAAGCTGGGTT**T**GAAATCGAAAAGCATGGTAATAAAGT-3’ (end of gene is bolded). The resulting PCR product was recombined into pDONR221 using the BP protocol as described by the manufacturer (Invitrogen). All other pENTR clones were previously described (8) or generated by the Pathogen Functional Genomic Resource Center.

For pKT129, *ct129* was amplified from *C. trachomatis* genomic DNA with primers 5’-TGGTGGGATCCT**G**AACACTATTTGCTAACA-3’ (*Bam*HI site underlined and start of gene bolded) and 5’-TTTTAGGTACCAT**T**GCAGACTCCTTCTCTGA-3’ (*Kpn*I site underlined and end of gene bolded). The resulting PCR product was digested and inserted into the corresponding *Bam*HI and *Kpn*I sites of pKT25.

For pKT144, *ct144* was amplified from *C. trachomatis* genomic DNA with primers 5’-TTCCCGGATCCT**A**CAACGCCAGATAATAAT-3’ (*Bam*HI site underlined and start of gene bolded) and 5’-ATTTAGGTACCAT**A**GGAACAACAGGTAGCCG-3’ (*Kpn*I site underlined and end of gene bolded). The resulting PCR product was digested and inserted into the corresponding *Bam*HI and *Kpn*I sites of pKT25.

For pKT471, *ct471* was amplified from *C. trachomatis* genomic DNA with primers 5’-ATTGTGGATCCT**G**AGAAGAGAGGCGTTATT-3’ (*Bam*HI site underlined and start of gene bolded) and 5’-AAGGCGGTACCAT**A**CGGAGAAAAATAACTCC-3’ (*Kpn*I site underlined and end of gene bolded). The resulting PCR product was digested and inserted into the corresponding *Bam*HI and *Kpn*I sites of pKT25.

All *att*L-flanked genes were introduced by recombination from pENTR clones into the corresponding BACTH-DEST plasmid using the LR protocol as described by the manufacturer (Invitrogen). For example, pENTR760 was recombined with pST25-DEST to generate pST760.

For pSO1 [P_BAD_::*CTrodZ*], *ct009* was amplified from *C. trachomatis* genomic DNA with primers 5’-TATATGGTACCTGATTAACTTTATAAGGAGGAAAAACAT**A**TGGGTAGAGCAAACCAGGGGAAT-3’ (*Kpn*I site underlined and start site bolded; also includes ribosome binding site) and 5’-ATCCCGTCGAC**T**TAGAAAAGGTTGAATAGATTCCCTAGCCACCAAATACAA-3’ (*Sal*I site underlined and stop site bolded). The resulting PCR product was digested and inserted into the corresponding *Kpn*I and *Sal*I sites of pBAD33 (10).

For pLP173 [P_lac_::*gfp-CTrodZ*], a portion of pSO1 [P_BAD_::*CTrodZ*] was amplified with primers 5'- GAGAGGCCATTACGGCCAGCGAACATGTCCACAAAGAG-3' and 5'- CATTGGCCGAGGCGGCCTTAGAAAAGGTTGAATAGATTCCC-3', and the 442 bp *Sfi*I fragment was used to replace the 2565 bp *Sfi*I fragment of pLP14 [P_lac_::*gfp-ponA*].

For pLP174 [P_lac_::*gfp-rodZ*], the 1027 bp *Sfi*I fragment of pCH371 [P_lac_::*t18-rodZ*] was used to replace the 2565 bp *Sfi*I fragment of pLP14 [P_lac_::*gfp-ponA*].

1. **Dautin, N., G. Karimova, A. Ullmann, and D. Ladant.** 2000. Sensitive genetic screen for protease activity based on a cyclic AMP signaling cascade in *Escherichia coli*. J. Bacteriol. **182**: 7060-7066.

2. **Bendezu, F. O., C. A. Hale, T. G. Bernhardt, and P. A. de Boer.** 2009. RodZ (YfgA) is required for proper assembly of the MreB actin cytoskeleton and cell shape in *E.coli*. EMBO J. **28:**193-204.

3. **Bernhardt, T. G., and P. A. de Boer.** 2004. Screening for synthetic lethal mutants in *Escherichia* *coli* and identification of EnvC (YibP) as a periplasmic septal ring factor with murein hydrolase activity. Mol. Microbiol. **52:**1255-1269.

4. **Bernhardt, T. G., and P. A. J. de Boer.** 2003. The *Escherichia* *coli* amidase AmiC is a periplasmic septal ring component exported via the twin-arginine transport pathway. Mol. Microbiol. **48:**1171-1182.

5. **Gerding, M. A., Y. Ogata, N. D. Pecora, H. Niki, and P. A. de Boer.** 2007. The trans-envelope Tol-Pal complex is part of the cell division machinery and required for proper outer-membrane invagination during cell constriction in *E. coli*. Mol. Microbiol. **63:**1008-1025.

6. **van den Ent, F., C. M. Johnson, L. Persons, P. de Boer, and J. Lowe.** 2010. Bacterial actin MreB assembles in complex with cell shape protein RodZ. EMBO J. **29:**1081-1090.

7. **Ouellette, S. P., E. Gauliard, Z. Antosova, and D. Ladant.** A Gateway® compatible bacterial adenylate cyclase-based two hybrid system. Env. Microbiol. Rep. *in press*

8. **Ouellette, S. P., G. Karimova, A. Subtil, and D. Ladant.** 2012. *Chlamydia* co-opts the rod-shape determining proteins MreB and Pbp2 for cell division. Mol. Microbiol. **85**: 164-178.

9. **Karimova, G., A. Ullmann, and D. Ladant.** 2001. Protein-protein interaction between *Bacillus stearothermophilus* tyrosyl-tRNA synthetase subdomains revealed by a bacterial two-hybrid system. J. Mol. Microbiol. Biotechnol. **3**: 73-82.

10. **Guzman, L.-M., D. Belin, M. J. Carson, and J. Beckwith.** 1995. Tight regulation, modulation, and high-level expression by vectors containing the arabinose P_BAD_ Promoter. J. Bacteriol. **177**: 4121-4130.
