# Supplementary figures and images for: Analysis of MreB interactors in Chlamydia reveals a RodZ homolog but fails to detect an interaction with MraY
Source: Front Microbiol. 2014 Jun 6;5:279. doi: 10.3389/fmicb.2014.00279 (PMC4047632; doi:10.3389/fmicb.2014.00279)

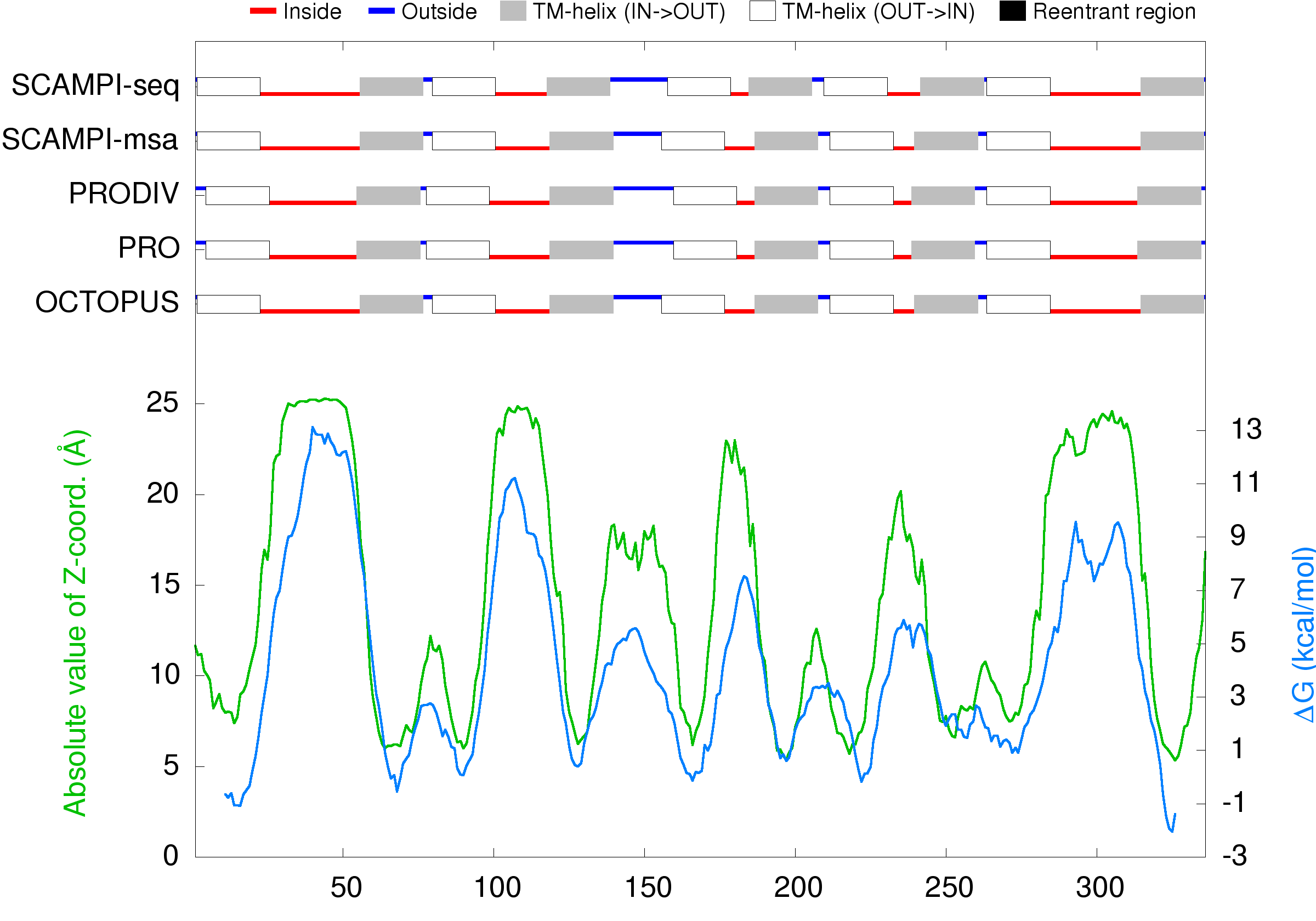

Supplement: Supplemental Figure S1 — TOPCONS membrane topological analysis of chlamydial MraY (Ct757). Note the presence of 10 TM domains with both the N- and C-termini located “outside.” [file Presentation1.ZIP › Supp Fig 1.TIF]

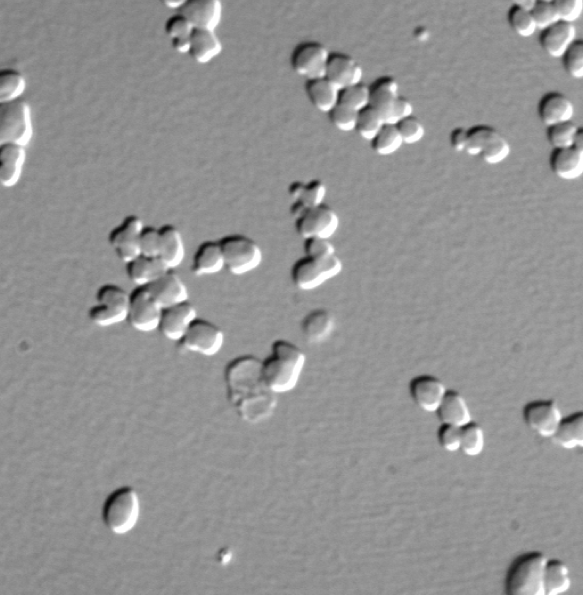

Supplement: Supplemental Figure S1 — TOPCONS membrane topological analysis of chlamydial MraY (Ct757). Note the presence of 10 TM domains with both the N- and C-termini located “outside.” [file Presentation1.ZIP › Supp Fig 2.TIF]

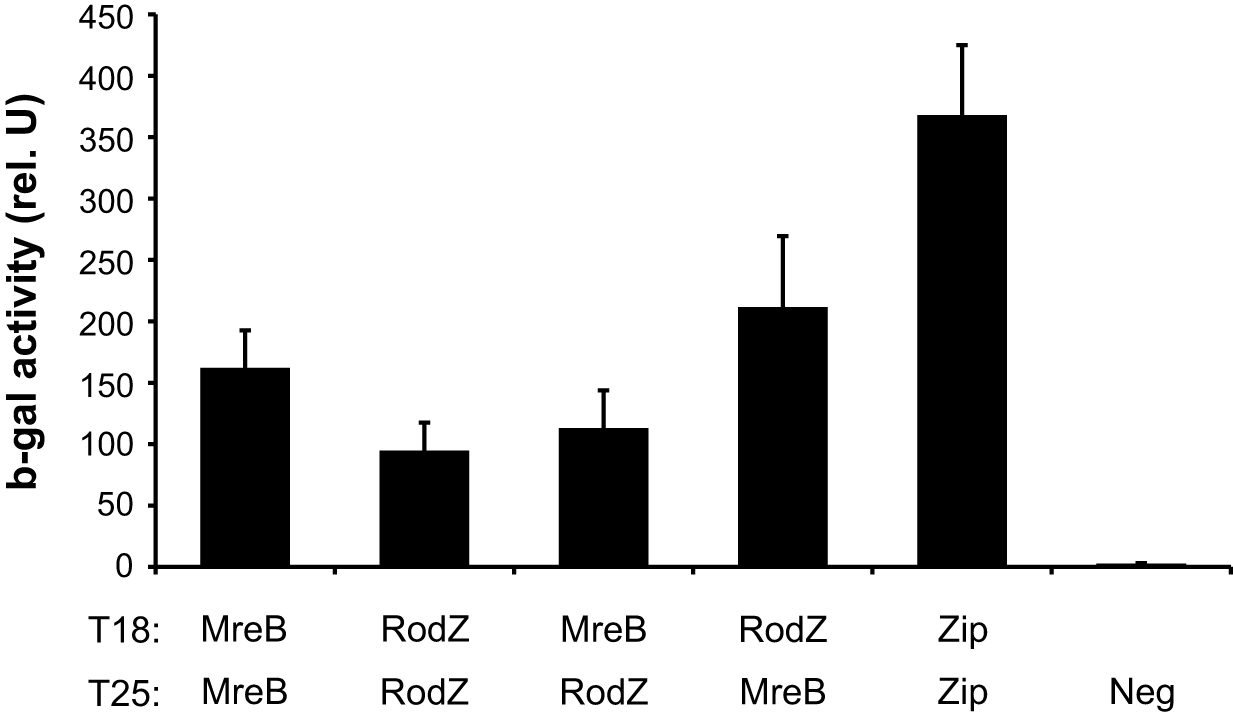

Supplement: Supplemental Figure S1 — TOPCONS membrane topological analysis of chlamydial MraY (Ct757). Note the presence of 10 TM domains with both the N- and C-termini located “outside.” [file Presentation1.ZIP › Supp Fig 3.TIF]
